# Two Surgeon General's reports on smoking and cancer: a historical investigation of the practice of causal inference

**DOI:** 10.1186/1742-7622-3-1

**Published:** 2006-01-10

**Authors:** Mark Parascandola, Douglas L Weed, Abhijit Dasgupta

**Affiliations:** 1 National Cancer Institute, Bethesda, Maryland, USA

## Abstract

**Background:**

The epidemiologic literature is replete with conceptual discussions about causal inference, but little is known about how the causal criteria are applied in public health practice. The criteria for causal inference in use today by epidemiologists have been shaped substantially by their use over time in reports of the U.S. Surgeon General on Smoking and Health.

**Methods:**

We reviewed two classic reports on smoking and health from expert committees convened by the US Surgeon General, in 1964 and 1982, in order to evaluate and contrast how the committees applied causal criteria to the available evidence for the different cancer sites at different time periods. We focus on the evidence for four cancer sites in particular that received detailed reviews in the reports: lung, larynx, esophagus and bladder.

**Results:**

We found that strength of association and coherence (especially dose-response, biological plausibility and epidemiologic sense) appeared to carry the most weight; consistency carried less weight, and temporality and specificity were apparently not applied at all in some cases. No causal claim was made for associations with a summary odds ratio of less than 3.0.

**Conclusion:**

Our findings suggest that the causal criteria as described in textbooks and the Surgeon General reports can have variable interpretations and applications in practice. While the authors of these reports may have considered evidential factors that they did not explicitly cite, such lack of transparency of methods undermines the purpose of the causal criteria to promote objective, evidence-based decision making. Further empirical study and critical examination of the process by which causal conclusions are reached can play an important role in advancing the practice of epidemiology by helping public health scientists to better understand the practice of causal inference.

## 

Forty years ago, the release of the Surgeon General's report on *Smoking and Health *marked a turning point in scientific and public health efforts to address the consequences of tobacco use for human health [1]. There had been earlier statements by the U.S. Surgeon General, in 1957 and 1959, declaring cigarette smoking to be an important cause of lung cancer [2,3], and there had been previous independent expert committees who reviewed the mass of available evidence and reached similar conclusions [4,5]. Nevertheless, the 1964 report became a landmark in the history of evidence-based public health because of its explicit use of pre-stated criteria for causal inference – consistency, strength, specificity, temporality, and coherence.

Essentially the same criteria are in wide use by epidemiologists today, and the report is frequently cited, along with Austin Bradford Hill's 1965 list of criteria [6], as a basis for causal inference in textbooks, literature reviews and consensus documents [7]. The history of scientific responses to the evidence linking cigarette smoking to various adverse health outcomes, particularly cancer, serves as a unique model case for understanding the evolution of contemporary epidemiologic practice. While the discipline of epidemiology has developed new quantitative methods and research tools in recent decades, the application of the causal criteria today has been shaped substantially by their early use in evaluating the evidence on smoking and health. However, while there has been considerable theoretical discussion of causal inference among epidemiologists over the years [8], there has been little effort to study how these criteria have been actually applied in practice. The most recent Surgeon General's report on the health consequences of smoking devotes an entire chapter to the changing causal language and conclusions across the reports, but the chapter does not include empirical analysis of *why *those conclusions changed (i.e. what were the key factors in the evidence that supported new, stronger causal conclusions?) [9].

In order to observe and understand how the causal criteria were applied to diverse bodies of evidence over time, we conducted a case study of the use of causal criteria in two reports of the Surgeon General on smoking and health, from 1964 and 1982. We reviewed the evidence available to the committees that authored these reports as well as the application of the causal criteria in the reports to the relationship between cigarette smoking and four cancers: lung, laryngeal, esophageal, and bladder. In addition to their historical interest, these reports allow us to observe how two expert panels with a common mandate apply identical, pre-stated inferential criteria to the same questions as the evidence accumulates over time. Additionally, this particular example allows us to focus on the process of causal inference because, while judgments about the relationship between smoking and these four cancers changed over time, each of these cancers has since been clearly linked to smoking [10].

## Committees and causal criteria

By the early 1960s there were already two statements from the Surgeon General and at least two consensus statements from groups of public health scientists concluding that the evidence was sufficient to claim that cigarette smoking was a cause of lung cancer [2,3]. In 1957, the Study Group on Smoking and Health, including members of the American Cancer Society, the American Heart Association, the National Cancer Institute (NCI) and the National Heart Institute concluded that "The sum total of scientific evidence establishes beyond a reasonable doubt that cigarette smoking is a causative factor in the rapidly increasing incidence of human epidermoid carcinoma of the lung." [11] Two years later, a follow up review prepared by a group of leading epidemiologists and biostatisticians (including Jerome Cornfield, William Haenszel, Michael Shimkin, E. Cuyler Hammond, Abraham Lilienfeld and Ernst Wynder) took account of the growing pool of cohort data and responded in substantial detail to various critiques that had been offered of the evidence. They admitted that the questions were not closed to further study, but they urged that "[t]he doctrine that one must never assess what has already been learned until the last possible piece of evidence [is in] would be a novel one for science." The fact that more could be learned did not preclude making judgments, and if it were not for the power of tobacco and the tobacco industry, the evidence "would be generally be regarded as beyond dispute." [12] Similarly, the Surgeon General released official statements on smoking and health in 1957 and 1959 declaring first that "the evidence is increasingly pointing in one direction: that excessive cigarette smoking is one of the causative factors in lung cancer" [2] and later that smoking is "the principle [sic] etiological factor in the increasing incidence of lung cancer."[3]

These reviews applied similar criteria to those applied in the 1964 Surgeon General's report. Robert Koch's postulates had previously provided a framework for identifying causes of infectious diseases, but those postulates, which required isolation of a parasite and inducing the disease in the laboratory, had limited application for environmental causes of chronic diseases. Thus, as the evidence linking smoking and lung cancer developed during the 1950s, public health scientists debated the requirements for demonstrating causation, and criteria proposed during these debates were later used in the 1964 report [7,13].

On June 1, 1961, the American Cancer Society, the American Heart Association, the American Public Health Association, and the National Tuberculosis Association sent a joint letter to President Kennedy asking for the appointment of a special commission to address the need for government action on smoking and health. They urged that the evidence of harm was sufficient to require government intervention [14,15]. However, health leaders in the Kennedy Administration were initially reluctant to take action, responding that disagreement remained over how much of the lung cancer burden could be attributed to smoking and could, in turn, be prevented by smoking reduction efforts [16-18]. Thus, Surgeon General Luther Terry decided that what was needed first was a comprehensive review of all the data on smoking and health by an expert advisory committee. While public health activists saw this move as another delaying tactic [19], Terry understood the value of an irreproachable view of the science. Thus, the committee would provide an "objective assessment of the nature and magnitude of the health hazard," but would not conduct new research or make any policy recommendations [1].

Selection of the committee members was a crucial part of the process to create an authoritative committee. The 10 committee members were selected from a list of about 150 physicians and biomedical scientists who had not taken a prior public position on the smoking and health issue. Major medical associations, volunteer public health organizations, the Tobacco Institute, the Food and Drug Administration, the Federal Trade Commission and the President's Office of Science and Technology were all given the opportunity to delete anyone from the list for any reason. President Kennedy himself promised that there would be no political interference with the committee's work [20]. None of the scientists who had conducted studies of smoking and health and been active in earlier debates, such as Cuyler Hammond and Ernst Wynder, ended up on the committee. Of the ten members, all but two were medical doctors. Their expertise included pathology, surgery, chemistry, cancer biology, internal medicine, pharmacology, statistics, and epidemiology. Given the importance that the epidemiologic evidence took on in the inquiry, it is important to note that the committee included only one epidemiologist and one biostatistician (Table 1) [1].

**Table 1:** 1964 Report: Members of the Advisory Committee to the U.S. Surgeon General

**Name and Degrees**	**Discipline**
Stanhope Bayne-Jones, M.D., LL.d.,	Bacteriology, Public Health
Walter J. Burdette, M.D., Ph. D.,	Surgery, Genetics
William G. Cochran, M.A.,	Statistics
Emmanuel Farber, M.D., Ph. D.,	Pathology
Louis F. Fieser, Ph. D.,	Chemistry
Jacob Furth, M.D.,	Cancer Biology
John B. Hickam, M.D.,	Internal Medicine (cardiopulmonary disease)
Charles LeMaistre, M.D.,	Internal Medicine (pulmonary diseases)
Leonard M. Schuman, M.D.,	Epidemiology
Maurice H. Seevers, M.D., Ph. D.,	Pharmacology

The committee members held their first meeting at the National Library of Medicine in Bethesda, Maryland, on November 9, 1962, with their deliberations under strict secrecy [21]. Outside communication with the committee members was controlled. Unfortunately, there is little remaining documentation of the committee's process and deliberations. Records of the Committee at the National Archives do not include formal meeting minutes that might characterize the group's discussions [22]. Accounts of the committee's deliberations and process are largely anecdotal [23]. Additionally, no minority report was produced.

The 1964 committee was unique both in its process and in the degree of attention given to it. Over the years, the process for producing Surgeon Generals' reports changed as well. The 1982 report, *The Health Consequences of Smoking: Cancer*, was assembled by an editorial committee of scientists, and individual chapters were authored by small groups of a few experts with multiple layers of review. The largest chapter, *Biomedical Evidence for Determining Causality*, was written by respiratory medicine specialist Richard Bordow and epidemiologist Abraham Lilienfeld [24]. Many of the authors and reviewers were actively working on research related to smoking and health at the time. By this time, the key challenges facing the scientists were not whether smoking was a cause of lung cancer, but the extent of its effects on both smokers and nonsmokers, the potential benefits of low tar cigarettes and the success of smoking cessation efforts. Nevertheless, the criteria for causality that were applied were the same as those applied in the 1964 report.

## Methods

Two reports of the Surgeon General on smoking and health that applied causal criteria to the four cancers at different points in time (1964 and 1982) [1,24] form the basis of our investigation. We chose these two reports because they applied the same causal criteria yet also represent a time period during which evidence linking smoking and some cancers was growing substantially. We chose to examine the evidence for the causal role of smoking in the development of cancer at four sites in particular for which there was adequate evidence available to apply the causal criteria: lung, larynx, esophagus, and bladder.

Both the 1964 and 1982 reports documented their evaluation of causality using the same five causal criteria: consistency, strength, specificity, temporal relationship, and coherence. Coherence was a broad category that assessed whether all the evidence makes sense taken together, including information about dose response, mortality trends, and biologic plausibility. Definitions given for these criteria are essentially the same in both reports, although the definitions provided in the 1982 report are more thorough. However, our interpretation of the application of the causal criteria relies largely on descriptions provided in the reports. Official records in the National Archives from the Surgeon General's Advisory Committee on Smoking and Health, which authored the 1964 report, did not yield additional details about the committee's causal reasoning. [25]

In both reports, cohort mortality and case-control study data were available for all cancers. In order to clearly summarize the case-control evidence available to the 1964 and 1982 committees, we conducted our own pooled analysis of the case-control data. While the two committees did not make use of formal, quantitative meta-analytic tools to assess the case control data, we do so for the purpose of representing the body of data in a straightforward manner to the reader who may not be as familiar with the data as the authors of the reports were. We follow the Surgeon General reports in discussing case-control and cohort data separately rather than pooling different types of studies together. The reports did not provide p-values or confidence intervals when summarizing data, and they did not consistently report whether or not positive results were statistically significant. We did not conduct a similar pooled analysis for the cohort data because some of these studies were not yet published at the time; although the Committee had access to the raw data for some studies, these were not available to us. However, the 1964 report included a simple unadjusted pooled analysis of cohort data upon which we draw.

We obtained all original research papers for the case-control studies cited in the reports as studies of cigarette smoking and the four cancers. We conducted meta-analyses on the published data from the case-control studies using the DerSimonian-Laird method [26]. We excluded the case-control studies that included only women, since the smoking habits for men and women were quite different at the time, and we excluded studies that did not report numbers of cases and controls and numbers of smokers and nonsmokers within these groups, as these did not allow calculation of an odds ratio; for example, some papers only reported percentages or ratios without providing the actual numbers used to calculate them. Seven out of 61 studies (11%) were excluded for insufficient data. The studies included employed a diverse range of data collection and reporting procedures to describe smoking status; in this study comparisons were made only between smokers and non-smokers. Estimates were not adjusted for age or other potential confounders.

Separate meta-analyses were conducted for each cancer site, namely lung, esophagus, larynx and bladder, and summary odds ratios and confidence intervals were obtained. Additionally, separate meta-analyses were conducted for bladder and esophageal cancer for those studies included in the 1964 report and those included in the 1982 report. Each of the six meta-analyses was summarized graphically by boxplots showing the summary odds ratio and confidence interval for each meta-analysis.

## Results

**Figure 1:**
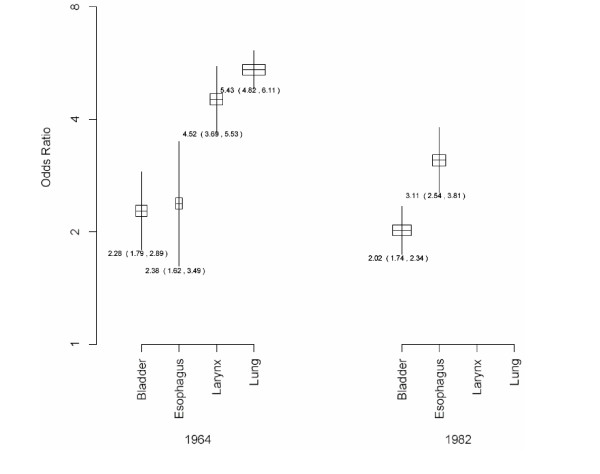
**A meta-analytic summary of the risk of cancer at four sites due to smoking, based on case-control studies from the Surgeon-General's reports of 1964 and 1982. **Each bar shows a 95% confidence interval of the summary odds ratio, with the point estimate and 95% confidence limits marked. The width of each bar is proportional to the total sample size of the studies included in the meta-analysis for that site.

The 1964 report concluded that there was sufficient evidence to regard cigarette smoking as a cause of both lung and laryngeal cancer (in men), but not of esophageal and bladder cancer. In contrast, the 1982 report concluded that cigarette smoking was a cause of esophageal cancer, but that the evidence remained insufficient for bladder cancer. Here we describe the evidence that was available for six causal assessments, focusing on comparisons between different types of cancer in the same report, and between reports for the same cancer type. These comparative analyses allow us to study how different bodies of evidence can result in different judgments about causation. The evidence cited in the reports for each of these causal assessments is described in Table 2. Results from our pooled analysis of the case-control studies are presented in Figure 1.

**Table 2:** Summary of evidence extracted from two Surgeon General reports

**1964**					**1982**	
	**Lung**	**Laryngeal**	**Esophageal**	**Bladder**	**Esophageal**	**Bladder**

**Consistency: CC**	24 studies/24 positive	10 studies/10 positive	6 studies/6 positive	4 studies/4 positive	9 studies/All positive	10 studies/8 positive
**Consistency: Cohort**	7 Studies/7 positive	7 studies/4 positive, 3 neutral	7 studies/6 positive, 1 negative	7 studies/6 positive, 1 negative	7 studies/All positive	8 studies/All positive
**Strength: CC**	5.43 (95% CI: 4.82 – 6.11)	4.52 (95% CI: 3.69 – 5.53)	2.38 (95% CI: 1.62 – 3.49)	2.28 (95% CI: 1.79 – 2.89)	3.11 (95% CI: 2.54 – 3.81)	2.02 (95% CI: 1.74 – 2.34)
**Strength: Cohort**	10.8 (range: 4.9 – 20.2)	5.4 (range: 1.5 – 13.1)	3.4 (range: .7 – 6.6)	1.9 (range: 0.9 – 6.0)	Range: 1.8 – 6.4	Prospective Studies: 1.4 – 2.9
**Specificity**	"90% [of lung cancer] is associated with cigarette smoking."(184) "the number of disease in which the ratios remain significantly high ... is not so great as to cast serious doubt on the causal hypothesis."(185)	Not stated	Not stated	Not stated	"Specificity ... is evidenced by substantial differences in the mortality ratios ... for esophageal cancer compared to other smoking related cancers."(96)	"lower order of ... specificity for bladder cancer than for cancers of the lung, larynx, oral cavity or esophagus suggests that factors other than smoking may be associated etiologically with bladder cancer."(108)
**Temporality**	"The early exposure to tobacco smoke and late manifestation of lung cancer among smokers, seem, at least superficially, to fulfill this condition." (185)	Not stated	Not stated	Not stated	"The temporal relationship of the association is supported by the prospective studies" "In addition, there are histological data suggesting that smoking predates premalignant and malignant transformation." (97)	"Evidence for the temporal relationship of the association is provided by the prospective studies" "Reliable histological studies of bladder epithelium in smokers compared with nonsmokers have not been reported."(110)
**Dose Response**	"In almost every study for which data were adequate" a gradient was observed with amount of smoking, duration, age when started, ex smokers, and inhalation	Gradient also observed with increased amounts of smoking and inhalation. "The parallelism with lung cancer, though not as complete because of a smaller amount of material, is remarkable."	Pooled cohort data revealed gradient for heavy versus moderate smokers. Gradient with amount smoked observed in only two of seven case control studies.	Pooled cohort data revealed gradient for heavy versus moderate smokers. Gradient with amount smoked observed in only two of four case control studies.	Dose response observed in retrospective and prospective studies	Modest dose response "however this relationship is not as strong as that noted between smoking and lung, laryngeal, oral, and esophageal cancers."(108)
**Biologic Plausibility: Animal/Lab Studies**	Application of tobacco smoke or condensates to lungs or tracheobronchial tree in animals failed to induce lung tumors (except possibly in dogs) [106, 248, 206, 224, 205a, 273, 274, 275, 29, 289]	"No attempts to induce carcinoma of the larynx by tobacco smoker or smoke condensates have been reported."(210)	"No attempts to induce carcinoma of the esophagus by tobacco smoker or smoke condensates have been reported."(217)	Three teams of investigators have studied bladder cancer in mice treated with tobacco tars, but findings were inconsistent and their significance unclear [177, 75, 295]	"There is experimental evidence that benzo[a]pyrene is able to penetrate the cell membranes of the esophageal epithelium," producing papillomas and carcinoma.(101)	Not stated
**Biologic Plausibility: Human Pathology**	Histopathologic changes in lungs of smokers in a controlled blind study of 402 male patients	Histopathologic changes in larynx of smokers(271)	Not Stated	Not Stated	Examination of autopsy tissue from 1,268 men showed atypical cells much more common in smokers	[see temporality above]
**Biologic Plausibility: Localization of cancer**	"Localization of cancer in relation to type of smoking"(188)	"Localization of lesions"(210)	Not Stated	Not States	Not Stated	Not Stated
**Coherence**	Time Trends Sex Differential Urban-rural differences Socio-economic differential	Time Trends Sex Differential	"Mortality from esophageal cancer in the United States had shown a tendency to rise slightly among whites in the last 30 years."(218)	"information is lacking for an intelligent discussion of the sex differential" "urban rural differential is virtually non-existent"(225)	Sex differential Lower mortality rates in low smoking populations (Mormons and 7^th ^Day Adventists) Lower risk in ex-smokers Smoking acts synergistically in combination with alcohol.	Sex differential Lower mortality rates in low smoking populations (Mormons and 7^th ^Day Adventists) Lower risk in ex-smokers Occupational exposures associated with bladder cancer
**Causal?**	**Yes**	**Yes**	**No**	**No**	**Yes**	**No**

Numbers in parentheses are references to page numbers in the Surgeon General reports

### Lung cancer

The initial interest into the potential carcinogenic effect of smoking focused on lung cancer, because lung cancer mortality rates were increasing dramatically. Thus, as explained in the 1964 report, far more studies had been conducted of lung cancer (and far more deaths observed) than for the other cancers. The 1964 Surgeon General's report discussed lung cancer first and devoted more than five times as much space to it than for any of the other cancers. Much of the discussion responded to specific criticisms that had been raised in prior scientific debates about the growing evidence on smoking and health [5].

The summary odds ratio from 25 case-control studies was 5.43 (95% CI: 4.82 – 6.11). All but two of the studies reported statistically significant positive associations, although the odds ratios for the individual case-control studies varied widely, from 0.8 to 35.4. The report provided a summary mortality ratio from the seven cohort studies of 10.8 (range: 4.9 – 20.2). Two prospective studies of tobacco workers that had not found any increased mortality among smokers were dismissed by the Surgeon General's committee because of "major defects". These studies, conducted by the American Tobacco Company, compared disease rates in a heavy smoking population of tobacco workers with those of the general population, thus being vulnerable to the "healthy worker" effect, and did not have individual level information on smoking status [27,28].

While the cohort studies linked cigarette smoking to numerous diseases, the committee concluded that the specificity criterion was met for lung cancer because the strength of association was so much greater than for other diseases, and because they reasoned that the majority of lung cancers were attributable to smoking (based on the magnitude of the relative risk) [[1], p. 184].

The report devoted more space to addressing coherence than to any other criterion for causal inference. Studies of mortality trends revealed a correlation between lung cancer death rates and cigarette consumption in different countries, a dramatic rise in lung cancer mortality following a World War I increase in cigarette consumption, and higher mortality in men and urban populations consistent with smoking patterns. Five of the cohort studies provided sufficient data to demonstrate a dose-response trend for increasing amounts of smoking. The plausibility of causation was enhanced by the fact that the lung is directly in the exposure pathway. Pathology studies of cellular changes in the tracheobronchial tree of male patients at autopsy revealed that an increasing amount of smoking correlated with a higher percentage of atypical cells, interpreted as precursors to lung cancer. Evidence from animal studies, however, was lacking. Application of tobacco tars to the lungs and trachea of animals in ten studies yielded results that were negative or of "uncertain significance".

### Contrast between laryngeal and lung cancers in 1964

While the 1964 report gave the most attention to lung cancer, a positive causal conclusion was also made for laryngeal cancer. The summary odds ratio from 10 case-control studies of smoking and laryngeal cancer, all statistically significantly positive, was 4.52 (95% CI: 3.69 – 5.53), and the summary mortality ratio from seven cohort studies was 5.4 (range: 1.5 – 13.1). Thus, studies of both lung and laryngeal cancers exhibited summary relative risks greater than 4.0 in both case-control and cohort studies. For laryngeal cancer, all of the case-control studies and four of seven cohort studies showed statistically significant positive associations. However, fewer data were available to evaluate coherence (including dose-response trends) for laryngeal cancer and smoking, as only 10 case-control studies had been conducted of laryngeal cancer (compared with 24 for lung cancer) and there were only a total of 75 laryngeal cancer deaths (compared with 1833 for lung cancer) in all the cohort studies. No mention was made of specificity and temporality for laryngeal cancer in the report.

### Contrast between laryngeal and esophageal cancers in 1964

Although the 1964 Committee concluded that smoking was a cause of laryngeal cancer, it did not ascribe causality for esophageal cancer. What accounted for this difference? The summary odds ratio from six case-control studies of smoking and esophageal cancer was 2.38 (95% CI: 1.62 – 3.49) and the report's summary mortality ratio from seven cohort studies was 3.4 (range: 0.7 – 6.6). The one negative result was dismissed by the Committee because it was based on only four deaths, too few to be a stable estimate. Although a dose-response trend was seen in only two of seven case-control studies, a trend was seen in the combined cohort study data. The report did not cite any human pathology or animal studies. Mortality statistics revealed a slight rise in esophageal cancer over the previous three decades. Compared with laryngeal cancer, the evidence for esophageal cancer, as described in the report, came from fewer studies, showed a lower level of strength, a similar if not slightly increased level of consistency, and less coherence. In particular, the report noted the "smallness" of the rise in esophageal cancer mortality following increased smoking prevalence and the limited dose-response data.

### Contrast between esophageal and bladder cancers in 1964

We looked at the similarities and differences in the evidence for esophageal and bladder cancer in 1964, when no causal relationship was inferred for either. For bladder cancer there were only four case-control studies, with a summary odds ratio of 2.28 (95% CI: 1.79 – 2.89), though all were statistically significantly positive. The report provided a summary mortality ratio from seven cohort studies of 1.9 (range: 0.9 – 6.0). Again, the one negative result was dismissed by the Committee because it was based on too few deaths to be a stable estimate. A dose-response trend was only seen in two of four case-control studies and in the combined cohort study data. Studies of bladder cancers in mice treated with tobacco tars were inconsistent, and no human pathology studies were cited. The report noted that mortality trends did not suggest any sex differential or urban-rural difference consistent with smoking patterns. No mention was made of localization of lesions in the report. Compared with esophageal cancer, the evidence for bladder cancer demonstrated a lower level of strength, consistency, and coherence.

### Esophageal cancer: 1964 and 1982

As a causal claim was made for esophageal cancer in 1982 but not in 1964, we examined the impact of the evidence accumulated between the two reports. The summary odds ratio for 1982, based on three more case-control studies (for a total of 9), was 3.11 (95% CI: 2.54 – 3.81), and all but two of the studies reported statistically significant positive associations. The most substantial change was the additional data to support coherence, including a human pathology study, laboratory studies showing that benzo[a]pyrene, a known tobacco carcinogen, could penetrate esophageal epithelial cells, and observations of higher mortality in men than women and reduced mortality in populations with low smoking rates (Mormons and 7^th ^Day Adventists). The 1982 report stated that the evidence supported specificity, because the mortality ratio for esophageal cancer differed from those of other smoking related cancers, and that temporality was supported by the availability of prospective studies and human pathology studies suggesting pre-cancerous lesions. The 1982 Committee declared smoking to be a cause of esophageal cancer, while the 1964 Committee had not made that claim.

### Bladder cancer: 1964 and 1982

Neither report made a causal claim for bladder cancer. What new evidence was available in 1982 that remained insufficient? By 1982 there were ten case-control studies (compared with four in 1964) with a summary odds ratio of 2.02 (95% CI: 1.74 – 2.34). Two of the six post-1964 studies failed to yield a statistically significant positive result; however, there were more positive studies than in 1964. There were additional data to support coherence, including observations of higher mortality in men than women and reduced mortality in populations with low smoking rates (Mormons and 7^th ^Day Adventists). The 1982 report described a lower order of specificity for bladder cancer compared with the three other cancers and cited the existence of other factors etiologically related with bladder cancer. The existence of prospective studies provided evidence of temporality.

### Contrast between esophageal and bladder cancers in 1982

A positive causal claim was made in 1982 for esophageal but not bladder cancer. What accounted for this difference? The strength of association was stronger for esophageal than for bladder cancer (pooled odds ratio of 3.11 versus 2.02 in case-control studies). Statistically significant positive associations with smoking were found in all epidemiologic studies of esophageal cancer (nine case-control and seven cohort), and eight of 10 case-control and all eight cohort studies of bladder cancer. Studies of the effects of benzo[a]pyrene on esophageal cells and studies of esophageal autopsy tissue were described, but no such studies were cited for bladder cancer. For both cancers, a sex differential in mortality rates consistent with smoking habits and lower mortality in low and ex-smoking populations were cited. The 1982 report also stated that the evidence for specificity and temporality of the association was weaker for bladder than for esophageal cancer, because of the existence of other causes of bladder cancer and the absence of human pathology studies showing pre-malignant lesions in smokers.

### How Were the causal criteria applied?

From the six case studies taken together, we can observe that strength of association and coherence (especially dose-response, biologic plausibility and consistency with general patterns of disease and exposure) appeared to carry the greatest weight in these causal inferences, while consistency, although still a factor, appeared to carry less weight (Table 1). Instances in which a positive causal claim was made were supported by larger odds ratios; 'causal' cases each had an odds ratio greater than 3.0, while odds ratios for 'non-causal' cases were each less than 3.0. However, consistency was not always stronger for 'causal' cases. For example, the epidemiologic evidence for bladder cancer in 1982 (16 of 18 studies positive), which did not result in a 'causal' judgment, was of equal or greater consistency to that for laryngeal cancer in 1964 (14 of 17 studies positive), which did result in a 'causal' judgment. Human pathology studies were available in all 'causal' cases, but not in any 'non-causal' cases. Dose-response data were consistently weaker in 'non-causal' relative to 'causal' cases (i.e. weaker dose-response trends and fewer studies demonstrating a dose-response trend).

Evidential support did not differ with regard to the availability of animal studies; in most cases, animal studies were either not reported or failed to yield consistent findings. Thus, while findings in animals were not important for determining whether or not a causal conclusion could be reached in these particular cases, we can still conclude that the absence of such evidence was not a barrier to causal inference. Indeed, overall biologic evidence, particularly from human pathology studies, seemed to play an essential role in causal inference in these case studies, even though the committees did not explicitly set out biologic plausibility as a separate criterion distinct from coherence. Despite their inclusion as causal criteria, temporality and specificity were not cited at all in 1964, except in the case of lung cancer. In 1982, the evidence for temporality and specificity was described as weaker for bladder cancer than for esophageal cancer.

## Discussion

We analyzed the causal inferences of two expert committees with regard to smoking and lung, laryngeal, esophageal, and bladder cancer, summarizing the evidence that was available for each and making comparisons between inferences. Strength of association and coherence appeared to carry the most weight in the committees' inferences, while consistency appeared to carry less weight. Based on the committees' accounts of their evaluation of the evidence, the criteria of temporality and specificity were not applied at all in some cases. Within the category of coherence, evidence from human pathology studies, localization of exposure, and mortality patterns were highlighted. While the causal criteria are typically linked with the discipline of epidemiology in particular, they were applied to a range of different types of data and by committees with a wide range of expertise; in fact, the 1964 committee included only one epidemiologist out of ten members.

Based on the reports, it appears that the two committees did not consistently apply the criteria they set out at the start of their inquiries. In particular, although temporality and specificity were included as criteria, they were not referred to at all in the 1964 report with the exception of lung cancer. These results add to previous findings that authors of review papers apply causal criteria inconsistently, often excluding or even altering criteria without giving reasons for these changes [29]. It is possible that some criteria were used implicitly in the process of causal inference in the reports of the Surgeon General, but that their application was not adequately described. For example, while localization of cancer in the exposure pathway was not specifically mentioned in either report with reference to esophageal or bladder cancer, it may have played a role (albeit not explicitly acknowledged in the reports) in the failure to reach a causal conclusion for bladder cancer. However, this ambiguity itself presents substantial difficulties for communicating to others the evidential basis for the committees' conclusions. The 1964 report is considered a landmark specifically because it applied explicit pre-stated criteria for causal inference; the ability of these criteria to help ensure objective, evidence-based decision making is limited if their application is not sufficiently transparent.

The process of synthesizing evidence and drawing causal conclusions does not occur in isolation, of course, but within a social and political context, and sometimes that context, particularly in the case of smoking and lung cancer in 1964, is highly charged. Historians and sociologists of science have presented evidence that extra-scientific values, such as ideology or personal gain, can influence how scientists interpret evidence [30,31]. Additionally, extra-scientific values have been acknowledged to play a role in evidence-based public health, accounting in part for why different expert groups sometimes reach divergent conclusions based on identical evidence [32]. Other studies of expert scientific committees have provided evidence that scientific disagreements can to some degree be explained by political and other factors [33-35].

Luther Terry attempted to minimize such influences by limiting the committee's purview to reviewing the science, excluding policy recommendations, and by carefully selecting committee members who would not be perceived to be in conflict. Nevertheless, it is reasonable to ask whether the tobacco industry, which was challenging the science and vigorously opposing tobacco regulation at the time [36], could have influenced the committee's actions. However, in the end the committee took a strong position on the evidence linking smoking with lung and laryngeal cancer. Both the 1964 and 1982 reports probably tended towards being conservative in their conclusions merely as a result of the group process. There were certainly other groups and influential individual scientists who had arrived at the same causal conclusions much earlier. However, tobacco industry opposition did not prevent authors of the reports of the Surgeon General from reaching causal conclusions based on the evidence. While tobacco industry efforts to criticize the science may have contributed to a more conservative stance overall, it seems unlikely that they would explain the differences in outcomes for different causal questions (i.e. lung versus bladder cancer).

The value of the causal criteria is that they provide explicit guidelines for evaluating evidence which, in turn, help reduce the influence of extra-scientific values and make inferences as transparent as possible. However, when such rules are applied inconsistently or without explicit descriptions, there is greater scope for extra-scientific values to influence causal conclusions. The Royal College of Physicians (RCP) had released its own expert committee review on smoking and health in 1962, which concluded that cigarette smoking is a cause of lung cancer but did not claim causation for any other type of cancer. However, the RCP did not cite any explicit guidelines for causal inference or provide a detailed analysis of the evidence for cancers other than lung cancer, making it difficult to assess why they reached a different conclusion for laryngeal cancer from the 1964 Surgeon General's committee [37]. While this study suggests that use of the causal criteria does not make inferences entirely transparent, application of the criteria is certainly a major advance over not employing any explicit inferential criteria. Efforts to further clarify the meaning and application of the causal criteria, as well as other methods for evaluating evidence, may have substantial benefits for evidence-based medicine. Additionally, because evidential synthesis involves evidence from a range of disciplines, not only epidemiology, other disciplines may benefit from further study of inferential practices as well.

We are not suggesting that the behavior of these committees be held up as a "gold standard" or model to be emulated. Indeed, the 1964 Surgeon General's report sets a very high threshold for inferring causality, perhaps too high for contemporary epidemiologic investigations of modest effects and complex gene-environment interactions. There are few situations where the evidence for causation is as overwhelming as it was for cigarette smoking and lung cancer in 1964. In the cases we studied, for example, no causal inferences were made where the strength of association was less than 3.0. At the same time, ecologic studies showing population level trends in cigarette use and cancer mortality played an important role in supporting the criterion of coherence, while such studies appear to be given less weight by epidemiologists since then [38].

The amount and type of evidence expected to support a causal inference can change over time, as new scientific methods become available and new disciplines develop. Historians have suggested that the level of proof required to demonstrate a causal link between smoking and lung cancer was especially high in 1964, because the methods of chronic disease epidemiology in use were new and not well understood by scientists in other disciplines [39,40]. By 1982, epidemiologic methods had become more widely used and the health effects of smoking were more widely accepted by scientists and clinicians. But in spite of this twenty year gap, the 1982 report still did not declare a causal link between cigarette smoking and bladder cancer, demonstrating that the causal criteria continued to play an essential role. Additionally, attitudes about the role of potential conflicts of interest have changed over time [41]. The 1964 committee explicitly excluded two epidemiologic studies conducted and funded by a tobacco company [27,28], arguing that these studies were vulnerable to the "healthy worker" bias because they compared disease rates among cigarette company workers with those of the general population. While the sponsorship of these studies was not raised as an issue, it likely would be today [42].

Why study an episode from the past like this? We maintain that further case studies like this one, both contemporary and historical, can raise new research questions and provide essential information to allow epidemiologists and other public health scientists to critically examine application of causal criteria today. Our findings suggest that the method of causal inference that has been articulated in textbooks and in the Surgeon General reports is incomplete, in that individual criteria and their joint application can be open to multiple interpretations [43]. Thus, there are elements of practice that can only be understood by observing how causal criteria are applied in real situations. For example, in carrying out systematic reviews, authors should clearly define and state the causal criteria they are applying and consider whether they are all to be given equal weight. Further studies investigating more diverse applications of the causal criteria could allow for broader generalizations about the type and level of evidence required in practice for causal inferences in evidence-based public health.

## Competing interests

The author(s) declare that they have no competing interests.
